# Chronic Limb Ischemia due to Thrombosis of an Aneurysmal Degeneration of the Autogenous Vein Graft: A Case Report

**DOI:** 10.1055/s-0042-1743199

**Published:** 2022-08-07

**Authors:** Chrysanthi P. Papageorgopoulou, Konstantinos M. Nikolakopoulos, Ioannis G. Ntouvas, Spyros Papadoulas

**Affiliations:** 1 Department of Vascular Surgery, University General Hospital of Patras, Patras, Greece; 2 Vascular Unit, Department of Surgery, General Hospital of Patras, Patras, Greece

**Keywords:** aneurysmal degeneration, great saphenous vein, graft

## Abstract

Nonanastomotic aneurysmal degeneration of a great saphenous vein graft is an unusual condition, despite the common use of this conduit in arterial reconstructions. Vein grafts are at risk of degenerative changes, but the real cause remains unknown. Postoperative graft surveillance with duplex ultrasound scanning is important for maintaining patency of the venous graft. We present a rare case of chronic limb ischemia due to partial thrombosis of an aneurysm of the great saphenous vein graft.

## Introduction

True nonanastomotic aneurysmal degeneration of great saphenous vein grafts is an unusual condition, despite the common and widespread use of this conduit in arterial reconstructions. 1 Vein grafts are at risk of degenerative changes, especially when used in the pediatric population, but the real cause remains unknown. It is well documented that, especially in the first 1 to 2 years after graft implantation, nearly one-third of autogenous vein grafts develop intrinsic lesions, as a result of intimal hyperplasia, which can lead to graft failure. 2 3 4 However, according to the literature, the mean time from graft implantation to aneurysmal degeneration may be up to 7 years. 5 Vein graft surveillance is critical to the long-term success of any arterial reconstruction. Postoperative graft surveillance with a program of ankle-brachial index determination and duplex ultrasound scanning is important for monitoring and maintaining patency of the venous graft.

## Case Presentation

A 67-year-old male patient presented to the emergency department with pain of the right lower extremity at rest, which started 24 hours earlier. The patient had a history of arterial hypertension and infrainguinal right saphenous vein bypass 11 years ago, due to severe claudication (Rutherford 3 category). For 2 months, the patient again suffered severe claudication, but he did not seek medical advice.

Physical examination revealed no palpable peripheral pulses and an ankle-brachial index of 0.49. Duplex ultrasound scanning and computed tomography angiography recognized the presence of aneurysmal degeneration of the proximal and middle thirds of the graft, with thrombus, turbulence, and elevated velocities consistent with luminal narrowing at the middle of the graft.

**Fig. 1:**
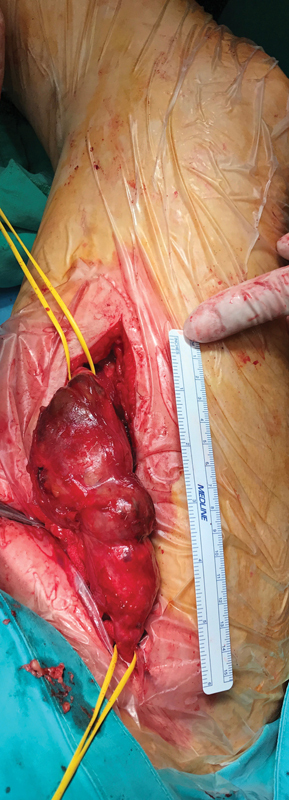
Aneurysmal degeneration of the proximal and middle third of the great saphenous vein graft.

**Fig. 2:**
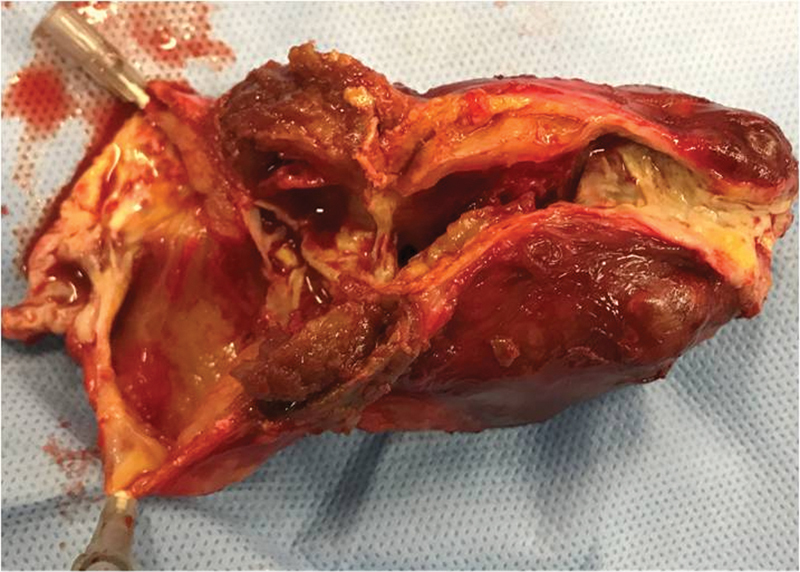
Mural thrombus had formed within the dilated portion of the graft just distal to an intrinsic stenosis.

**Fig. 3:**
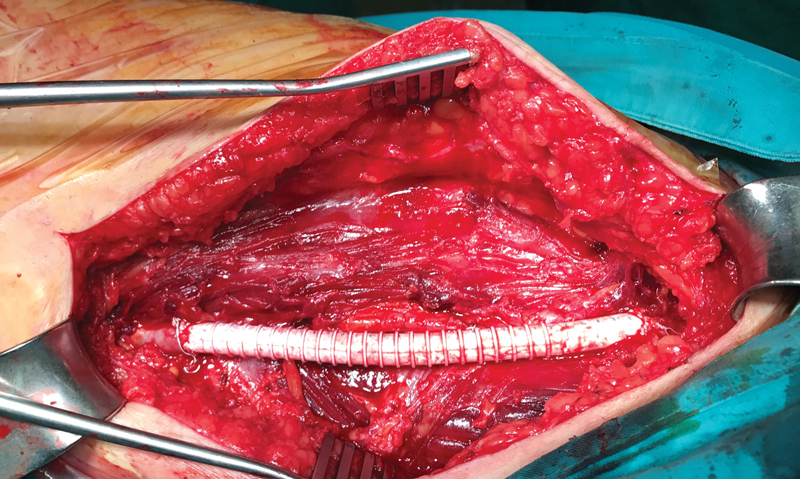
Complete replacement of the aneurysmal part of the venous graft with a prosthetic graft was performed.

Aneurysmectomy ( Fig. 1 ) and complete replacement of the aneurysmal part of the venous graft with a prosthetic graft was performed under general anesthesia. Thrombus had formed within the dilated portion of the graft just distal to an intrinsic stenosis, as mural thrombus tends to do in an arterial aneurysm ( Fig. 2 ). An 8 mm prosthetic “GORE-TEX Vascular Graft ( Fig. 3 ) was used because the contralateral great saphenous vein was unsuitable for use, due to small caliber, based on the preoperative duplex mapping.

Postoperative recovery was uneventful, without infection or hematoma development. The patient was discharged on the third postoperative day with palpable peripheral pulses. At the 6-month follow-up, the patient remains asymptomatic, with an ankle-brachial index around 0.96 and no sign of claudication or wound complication.

## Discussion

Although true nonanastomotic aneurysms of an infrainguinal vein graft are rarely reported in the literature, a recent publication 6 suggested that the incidence may be as high as 8.8%. Advanced atherosclerotic changes with extensive intimal hyperplasia, subintimal cholesterol deposits, and ulcerations can be seen in the wall of the aneurysmal vein graft. Atherosclerosis has been considered to be the main cause of aneurysm formation in vein grafts, but additional factors like systemic dilation diathesis, venous graft varicosities, infection, and poststenotic dilatation should be further investigated. 7

Aneurysmal dilatations in vein grafts may rupture or develop thrombosis, causing hemorrhage or acute limb ischemia that require surgical treatment on an urgent basis. However, open repair of aneurysmal vein grafts can be challenging because of extensive scar tissue that leads to a high rate of wound infection. Despite the difficulties of reoperation, open repair remains the cornerstone of treatment in these cases. According to published literature, endovascular treatment has been used in two cases to treat dilated venous bypass grafts, 8 but distal embolization and malperfusion can occur and prompt intervention through open or endovascular procedure may become necessary.

Vein graft surveillance is the key to long-term success of infrainguinal bypasses. Postoperative graft surveillance with a program of ankle-brachial index determination and duplex ultrasound scanning is important for maintaining patency. Lesion progression may indicate early intervention for the rescue of the venous grafts.

In conclusion, true nonanastomotic aneurysms of an infrainguinal vein graft are rare, and their etiology remains unknown. These can lead to graft thrombosis, rupture, distal embolization, and skin ulceration due to continuous pressure. Therefore, a graft surveillance program of ankle-brachial index determination and duplex ultrasound scanning is mandatory, as early diagnosis and intervention can minimize the potential late complications.
